# The Combination of Biochar and *Bacillus subtilis* Biological Agent Reduced the Relative Abundance of Pathogenic Bacteria in the Rhizosphere Soil of *Panax notoginseng*

**DOI:** 10.3390/microorganisms12040783

**Published:** 2024-04-12

**Authors:** Yingjie Zhou, Yanwei Liu, Siwen Li, Qiliang Yang

**Affiliations:** 1 Faculty of Modern Agricultural Engineering, Kunming University of Science and Technology, Kunming 650500, China; zhouyingjie@stu.kust.edu.cn (Y.Z.); 20110170@kust.edu.cn (Y.L.); 20222214040@stu.kust.edu.cn (S.L.); 2 Yunnan Provincial Field Scientific Observation and Research Station on Water-Soil-Crop System in Seasonal Arid Region, Kunming University of Science and Technology, Kunming 650500, China; 3 Yunnan Provincial Key Laboratory of High-Efficiency Water Use and Green Production of Characteristic Crops in Universities, Kunming University of Science and Technology, Kunming 650500, China

**Keywords:** *Panax notoginseng*, biological agent, *Bacillus subtilis*, rhizosphere soil fungal community

## Abstract

In the continuous cropping of *Panax notoginseng*, the pathogenic fungi in the rhizosphere soil increased and infected the roots of *Panax notoginseng*, resulting in a decrease in yield. This is an urgent problem that needs to be solved in order to effectively overcome the obstacles associated with the continuous cropping of *Panax notoginseng*. Previous studies have shown that *Bacillus subtilis* inhibits pathogenic fungi in the rhizosphere of *Panax notoginseng*, but the inhibitory effect was not stable. Therefore, we hope to introduce biochar to help *Bacillus subtilis* colonize in soil. In the experiment, fields planted with *Panax notoginseng* for 5 years were renovated, and biochar was mixed in at the same time. The applied amount of biochar was set to four levels (B0, 10 kg·hm^−2^; B1, 80 kg·hm^−2^; B2, 110 kg·hm^−2^; B3, 140 kg·hm^−2^), and *Bacillus subtilis* biological agent was set to three levels (C1, 10 kg·hm^−2^; C2, 15 kg·hm^−2^; C3, 25 kg·hm^−2^). The full combination experiment and a blank control group (CK) were used. The experimental results show that the overall *Ascomycota* decreased by 0.86%~65.68% at the phylum level. *Basidiomycota* increased by −73.81%~138.47%, and *Mortierellomycota* increased by −51.27%~403.20%. At the genus level, *Mortierella* increased by −10.29%~855.44%, *Fusarium* decreased by 35.02%~86.79%, and *Ilyonectria* increased by −93.60%~680.62%. *Fusarium* mainly causes acute bacterial wilt root rot, while *Ilyonectria* mainly causes yellow rot. Under different treatments, the Shannon index increased by −6.77%~62.18%, the Chao1 index increased by −12.07%~95.77%, the Simpson index increased by −7.31%~14.98%, and the ACE index increased by −11.75%~96.12%. The good_coverage indices were all above 0.99. The results of a random forest analysis indicated that *Ilyonectria*, *Pyrenochaeta*, and *Xenopolyscytalum* were the top three most important species in the soil, with MeanDecreaseGini values of 2.70, 2.50, and 2.45, respectively. *Fusarium*, the primary pathogen of *Panax notoginseng*, ranked fifth, and its MeanDecreaseGini value was 2.28. The experimental results showed that the B2C2 treatment had the best inhibitory effect on *Fusarium*, and the relative abundance of *Fusarium* in *Panax notoginseng* rhizosphere soil decreased by 86.79% under B2C2 treatment; the B1C2 treatment had the best inhibitory effect on *Ilyonectria*, and the relative abundance of *Ilyonectria* in the *Panax notoginseng* rhizosphere soil decreased by 93.60% under B1C2 treatment. Therefore, if we want to improve the soil with acute Ralstonia solanacearum root rot, we should use the B2C2 treatment to improve the soil environment; if we want to improve the soil with yellow rot disease, we should use the B1C2 treatment to improve the soil environment.

## 1. Introduction

*Panax notoginseng* is a perennial herb of Araliaceae. At present, the main producing areas are located in Yunnan and Guangxi. The planting area and yield of *Panax notoginseng* in Wenshan County, Yunnan Province, account for more than 95% of the total planting area of *Panax notoginseng* in China [1,2]. Large-scale planting has not only played an essential role in local economic growth but has also created many jobs. *Panax notoginseng* is a valuable traditional Chinese medicine with high medicinal value and plays a vital role in treating cardiovascular diseases [3,4,5]. *Panax notoginseng* is the primary raw material in many proprietary Chinese medicines, such as Yunnan Baiyao, Pian Tsai Yi, and compound Danshen tablets. With the increase in people’s healthcare awareness, the market demand for *Panax notoginseng* also increases yearly. However, due to the particularity of the growth cycle and growth environment of *Panax notoginseng*, the plant is vulnerable to diseases in the growth process [6]. With the increase in planting years, the colony structure in the soil of *Panax notoginseng* became out of balance, which led to the increase in disease species and disease degree [7]. At present, root rot is one of the most common diseases restricting *Panax notoginseng* yield [8]. In previous studies, it was found that root rot disease was mainly due to the fact that with the increase in planting years, the related pathogenic bacteria in the rhizosphere soil of *Panax notoginseng* gradually became the dominant colonies, and then infected the roots [9]. In addition, black spot, round spot, and anthracnose are all common diseases that occur in the planting process of *Panax notoginseng* [1,2]. These diseases seriously restrict the healthy development of the *Panax notoginseng* industry. Therefore, it is essential and urgent to explore field management measures to alleviate soil environmental stress in the rhizosphere of *Panax notoginseng*.

Previous studies mainly focused on reducing the occurrence of *Panax notoginseng* diseases and increasing the yield by establishing a reasonable field water and fertilizer management system [10,11]. Such a management system has also been proven to reduce the incidence of *Panax notoginseng* diseases significantly [12,13]. However, with the increase in planting years, the imbalance in the colony structure in the soil of *Panax notoginseng* has become more serious, and the effect of simple water and fertilizer management is weaker [14,15]. Therefore, introducing more effective means of controlling the rhizosphere soil colony is one of the most important ways to ensure the health of *Panax notoginseng*. Many microorganisms can promote plant growth, and the bacteria that promote plant growth through beneficial effects on the plant rhizosphere are called plant-growth-promoting rhizobacteria (PGPR) [16]. Previous studies have shown that *Bacillus subtilis* helps plants resist pathogens by producing volatile organic compounds and secreting polysaccharides and iron ion carriers [17,18]. At present, the biological products related to *Bacillus subtilis* are widely used. However, the inhibitory effect could be more stable, and we posit that it is challenging to colonize *Bacillus subtilis* because of the complex soil environment. Biochar is a stable carbon-rich product formed by the pyrolysis of agricultural and forestry wastes under an anoxic environment [19]. Previous research results have shown that after biochar is applied to soil, in a particular range, the number and activity of soil microorganisms increase significantly with the amount of biochar applied. It is generally believed in academic circles that this is because the uniform and dense pores of biochar in the soil can provide a suitable environment for microorganisms to inhabit and reproduce, thus reducing the survival competition among microorganisms [20,21,22,23].

The aims of this research were as follows: (1) to explore the fungal structure in the rhizosphere soil of multi-cropping *Panax notoginseng*; (2) to examine the effects of *Bacillus subtilis* on the fungal community structure and related pathogens in the rhizosphere soil of continuous-cropping *Panax notoginseng* under the condition of carbon addition; and (3) to understand the response of different fungi in the rhizosphere soil of *Panax notoginseng* to *Bacillus subtilis* biological agents under adding biochar.

## 2. Materials and Methods

### 2.1. Experimental Site and Experimental Design

**Figure 1:**
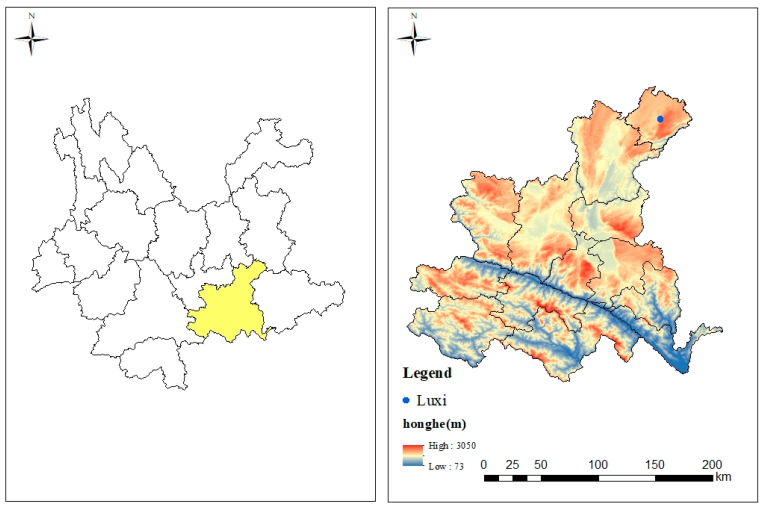
Location of pilot areas.

The experimental site is located in the research and demonstration base of key technologies of *Panax notoginseng* for water control, pollution reduction, quality improvement, and efficiency improvement in Luxi County, Yunnan Province, China (24°26′ N, 103°34′ E, altitude 1945 m) (Figure 1). The experimental treatment was the addition level of *Bacillus subtilis* biological preparation and biochar. The addition of *Bacillus subtilis* biological agents was set at three levels: low addition level (10 kg·hm^−2^, C1), medium addition level (15 kg·hm^−2^, C2) and high addition level (25 kg·hm^−2^, C3). There were four levels of biochar addition, namely, no addition of biochar (0 kg·hm^−2^, B0), low addition level (80 kg·hm^−2^, B1), medium addition level (110 kg·hm^−2^, B0) and high addition level (140 kg·hm^−2^, B3). At the same time, a control treatment without the addition of *Bacillus subtilis* and biochar was set up (CK). The full combination experiment was used in the experiment, with a total of 13 treatments (Table 1). The biological preparation of *Bacillus subtilis* was produced by Shandong Shuoyuan Ecological Agriculture Development Co., Ltd. The biochar used in this study was a black substance produced by using branches as raw materials and treated at high temperature (500–600 °C) under anoxic conditions. Biochar was purchased from Yunnan Weixin Agricultural Technology Co., Ltd. (Kunming, China). Each experimental small area was 4 m long and 1.5 m wide, and all plots used independent micro-sprinkler irrigation systems. According to local planting experience and previous research results [11], the amount of single irrigation is 10 mm, once every 7 days, and the total amount of irrigation is 480 mm. A large number of element water-soluble fertilizers (N:P_2_O_5_:K_2_O = 1:1:2) produced by Sichuan Shifang Demi Industrial Co., Ltd. (Shifang, China) were selected for use, and the fertilization was completed by the integrated technology of water and fertilizer. *Panax notoginseng* was planted in the experimental community for five consecutive years, from 2018 to 2022. The community was renovated after harvest in October 2022. *Panax notoginseng* seedlings were purchased from local farmers in Luxi. Planting was completed in November 2022, with each plot separated by 10 cm and covered with 1 cm thick pine needles on each low surface after planting. Sampling was carried out in November 2023, and three *Panax notoginseng* plants were randomly selected from each plot, gently shaking the *Panax notoginseng* plants, and the soil still left on the roots of *Panax notoginseng* was the rhizosphere soil. The rhizosphere soil of *Panax notoginseng* was collected and stored in liquid nitrogen at −80 °C for further sequencing experiment. Sequencing service was provided by Shanghai Ouyi Biology (Illumina Inc., San Diego, CA; OE Biotech Company; Shanghai, China).

### 2.2. DNA Extraction and Amplification

Total genomic DNA was extracted using MagPure Soil DNA LQ Kit (Magan), following the manufacturer’s instructions. DNA concentration and integrity were measured with NanoDrop 2000 (Thermo Fisher Scientific, Waltham, MA, USA) and agarose gel electrophoresis. Extracted DNA was stored at −20 °C until further processing. The extracted DNA was used as template for PCR amplification of fungal ITS genes with the barcoded primers and Takara Ex Taq (Takara). For fungal diversity analysis, the ITS1 variable regions of ITS genes was amplified with universal primers ITS1F (5′-CTTGGTCATTTAGAGGAAGTAA-3′) and ITS2 (5′-GCTGCGTTCTTCATCGATGC-3′) [24].The amplicon quality was visualized using agarose gel electrophoresis. The PCR products were purified with AMPure XP beads (Agencourt) and amplified for another round of PCR. After purification with the AMPure XP beads again, the final amplicon was quantified using Qubit dsDNA Assay Kit (Thermo Fisher Scientific, USA). The concentrations were then adjusted for sequencing. Sequencing was performed on an Illumina NovaSeq 6000 with 250 bp paired-end reads (Illumina Inc., San Diego, CA; OE Biotech Company; Shanghai, China).

### 2.3. Bioinformatic Analysis

The library sequencing and data processing were conducted by OE biotech Co., Ltd. (Shanghai, China). Raw sequencing data were in FASTQ format. Paired-end reads were then preprocessed using Cutadapt software (version 2.6) to detect and cut off the adapter. After trimming, paired-end reads were filtered for low-quality sequences, denoised, and merged, and the chimera reads were detected and removed using DADA2 [25] with the default parameters of QIIME2 [26] (2020.11). Finally, the software output the representative reads and the ASV abundance table. The representative read of each ASV was selected using QIIME2 package. All representative reads were annotated and blasted against Unite database using q2-feature-classifier with the default parameters.

QIIME2 software (Version 2022.2.0-1) was used for alpha and beta diversity analysis. The microbial diversity in samples was estimated using the alpha diversity that included the Chao1 index and Shannon index. The Bray–Curtis distance matrix performed by R package [27] (Version 3.5.1) was used for Bray–Curtis principal coordinate analysis (PCoA) to estimate the beta diversity. Then, the R package was used to analyze the significant differences between different groups using ANOVA statistical test.

### 2.4. Statistical Analysis

The Alpha diversity index of soil fungi was analyzed by SPSS22 software (version 22.0.0.0.202). The vegan package in R (Version 3.5.1) was used to draw heatmaps and the composition of fungal communities for principal coordinate analysis (PCoA); random forest analysis was performed using the R package randomForest (Version 4.7-1.1).

## 3. Results

### 3.1. Community Structure of Fungi in Rhizosphere Soil of Panax notoginseng

**Figure 2:**
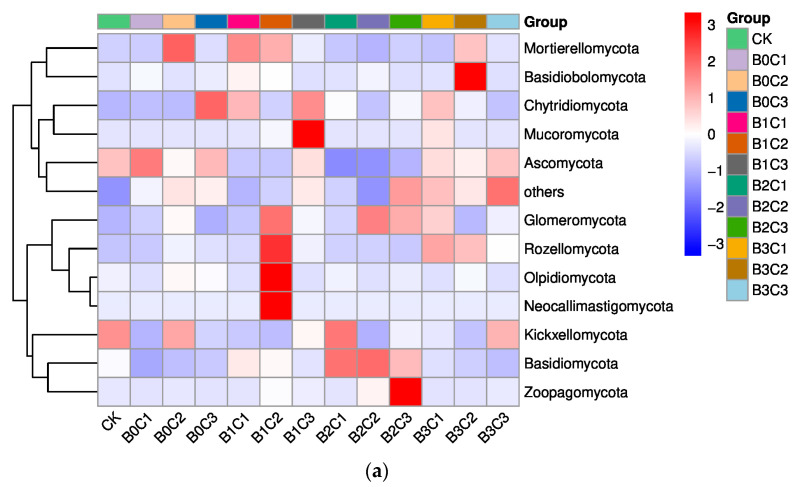

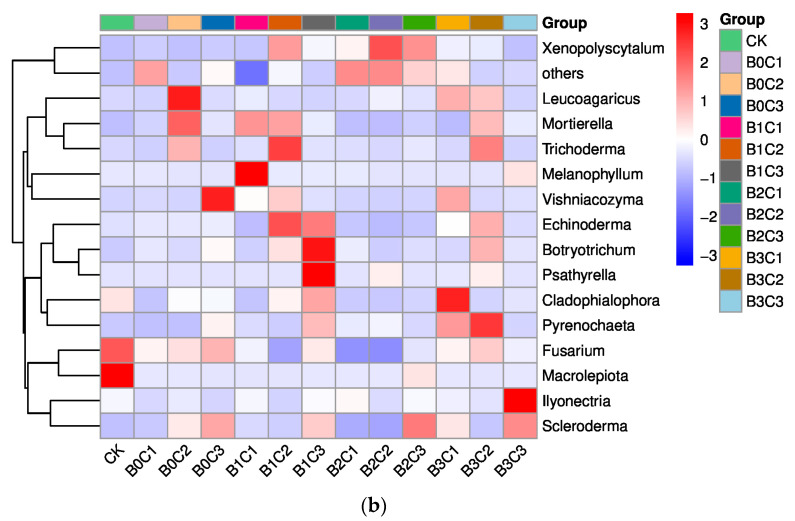
Heatmap diagram of the community structure of each group at the phylum level. Plotting a heatmap requires species relative abundance information. At the phylum level, we selected the top 15 relative abundance species for heatmap drawing in groups. The abscissa is sample information, the right ordinate is species annotation information, and the left ordinate is clustering information. (**a**) Heatmap diagram of the community structure of each group at the phylum level; (**b**) heatmap diagram of the community structure of each group at the genus level.

The species formation heatmap of the top 10 species was selected at the phylum level (Figure 2a). The results showed that *Ascomycota*, *Basidiomycota* and *Mortierellomycota* were the top three species in the rhizosphere soil of perennial *Panax notoginseng*. Compared with the CK treatment, the relative abundance of *Ascomycota* decreased by 0.86%~65.68% in the treatments with biochar addition. Specifically, it decreased by 0.86% under the B3C3 treatment and by 65.68% under the B2C2 treatment. In the treatments without biochar addition, the relative abundance of *Ascomycota* varied from −20.10% to 26.00%, specifically increasing by 26.00% in the treatment with B0C1 and decreasing by 20.10% in the treatment with B0C2. Compared with the CK treatment, the relative abundance of *Basidiomycota* varied from −53.03% to 138.47% in the treatments with biochar addition, specifically decreasing by 53.03% under the B3C3 treatment and increasing by 138.47% under the B2C2 treatment. In the treatments without biochar addition, the relative abundance of *Basidiomycota* decreased by 43.38%~73.81%, specifically by 73.81% in the B0C1 treatment and by 43.38% in the B0C3 treatment. Compared with the CK treatment, the relative abundance of *Mortierellomycota* varied from −51.27% to 322.11% in the treatments with biochar addition, specifically decreasing by 51.27% under the B2C2 treatment and increasing by 322.11% under the B1C1 treatment. In the treatments without biochar addition, the relative abundance of *Mortierellomycota* varied from −6.23% to 403.20%, specifically decreasing by 6.23% in the treatment with B0C1 and increasing by 403.20% in the treatment with B0C2.

The species generation heatmap of the top 15 species was selected at the genus level (Figure 2b). The results showed that *Mortierella*, *Fusarium* and *Melanophyllum* were the top three species in the rhizosphere soil of *Panax notoginseng*. Compared with the CK treatment, the relative abundance of *Mortierella* varied from −10.29% to 659.91% in the treatments with biochar addition. Among them, the relative abundance of *Mortierella* decreased by 10.29% under the B3C1 treatment and increased by 659.91% under the B1C1 treatment. In the treatments without biochar addition, the relative abundance of *Mortierella* increased by 81.33%~855.44%, specifically by 81.33% in the treatment with B0C1 and by 855.44% in the treatment with B0C2. Compared with the CK treatment, the relative abundance of *Fusarium* decreased by 35.02%~86.79% in the treatments with biochar addition, specifically by 35.02% under the B3C2 treatment and by 86.79% under the B2C2 treatment. In the treatments without biochar addition, the relative abundance of *Fusarium* decreased by 27.75% under the B0C3 treatment and by 48.31% under the B0C1 treatment. Compared with the CK treatment, the relative abundance of *Melanophyllum* varied from −100.00% to 18210.46% in the treatments with biochar addition. Among them, *Melanophyllum* was not detected under the B2C2 and B2C3 treatments, and its relative abundance increased by 18210.46% under the B1C2 treatment. In the treatments without biochar addition, the relative abundance of *Melanophyllum* varied from −100.00% to 68.33%, increasing by 68.33% under the B0C3 treatment and decreasing by 100.00% under the B0C1 treatment.

### 3.2. Fungal Diversity in Rhizosphere Soil

#### 3.2.1. Alpha Diversity Analysis

The results for the Alpha diversity index of the rhizosphere soil fungal community of *Panax notoginseng* (Table 2) showed that an average of 189 ASVs were detected in the CK treatment. Compared with the CK treatment, the number of ASVs increased significantly in the B0C3, B1C2, B3C1 and B3C2 treatments, but did not reach a significant level in other treatments. The Chao1 index ranged from −12.07% to 95.77%. The Chao1 index was used to estimate the number of ASVs in the soil, and the calculated result was close to the number of ASVs, so the significance was consistent with that of ASVs. The overall variation range of the Shannon index was −6.77%~62.18%; this index decreased significantly only under the B0C1 treatment. The Shannon index was consistent with the treatment with biochar addition and only slightly decreased under the B2C2 treatment. Under the B3C1 treatment, the average value of the Shannon index was 6.62, which was the maximum value in all treatments. Compared with the CK treatment, the Simpson index varied from −7.31% to 14.98% under different treatments, decreasing significantly only under the B0C1 and B2C2 treatments and increasing under the other treatments. The Simpson index under the B1C3 treatment was 0.98, the highest of all treatments. Compared with the CK treatment, the overall variation range of the AEC index was between −11.75% and 96.12%; its average value under the B1C1 treatment was 166.55, which was the lowest of all treatments. Under the B1C2 treatment, the ACE index was 370.14, the maximum value in all treatments. The good_coverage index was higher than 0.99, which indicated that the detection coverage could accurately reflect the actual soil condition.

**Figure 3:**
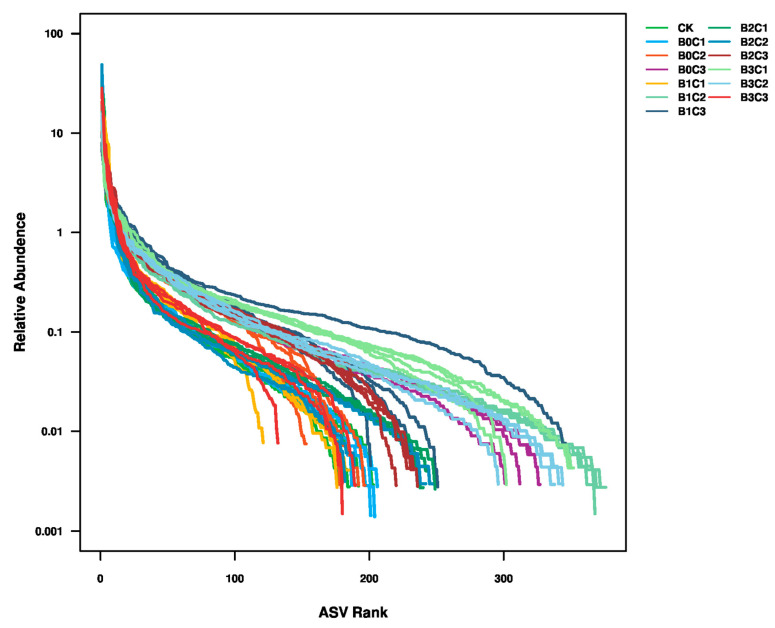
Rank abundance curve. Values on the abscissa are sorted by ASV according to the number of sequences they contain. Number on the abscissa indicate rankings of ASV abundance in the sample. For example, “300” indicates the corresponding relative abundance of the 300th ASV along the ordinate in the sample.

Rank abundance (Figure 3) was used to simultaneously explain the richness and uniformity of the species contained in the sample. The results showed that the species richness in the rhizosphere soil of *Panax notoginseng* was lower under the CK, B1C1 and B3C3 treatments, but increased under the B0C3, B1C2, B3C1 and B3C2 treatments.

#### 3.2.2. Beta Diversity Analysis

**Figure 4:**
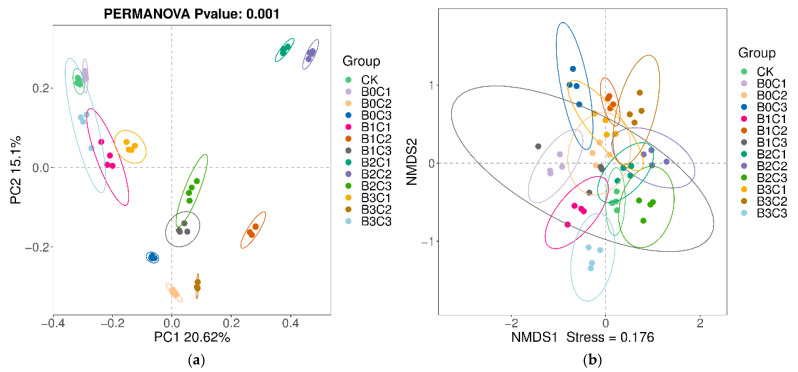
PCoA analysis and NMDS analysis. (**a**) PCoA analysis based on Bray–Curtis. Abscissa (PC1) and ordinate (PC2) are the two main coordinates with the greatest degree of explanation for the differences between samples. The same color is the same grouping, a point is a sample, and similar samples gather together. (**b**) NMDS analysis based on binary-jaccard. Abscissa (NMDS1) and ordinate (NMDS2) are two sort axes, each point in the graph represents a sample, the same color is the same grouping, similar samples will gather together. If the difference between samples is large, they will be farther apart in the diagram.

Beta diversity was used to compare the differences among different grouping samples. We used PCoA analysis based on Bay–Curtis (Figure 4a) to show the differences in the rhizosphere soil of *Panax notoginseng* treated with different biochar and biological agents. The results show that the degree of explanation of difference by PC1 was 20.62%. The degree of explanation of difference by Personality PC2 was 15.10%. The total explanation of the difference between PC1 and PC2 was 35.72%. Under different treatments, intra-group repetition can be better aggregated, showing little difference within the group, and the collected samples can objectively reflect the actual situation in the soil. There was no aggregation among the groups under different treatments, which indicated that the community structure in the rhizosphere soil of *Panax notoginseng* was changed by adding biological agents and biochar, resulting in differences among different treatments. The results of NMDS analysis based on binary-jaccard (Figure 4b) also showed that the Beta diversity in the rhizosphere soil of *Panax notoginseng* changed accordingly with the different application rates of biological agents and biochar.

**Figure 5:**
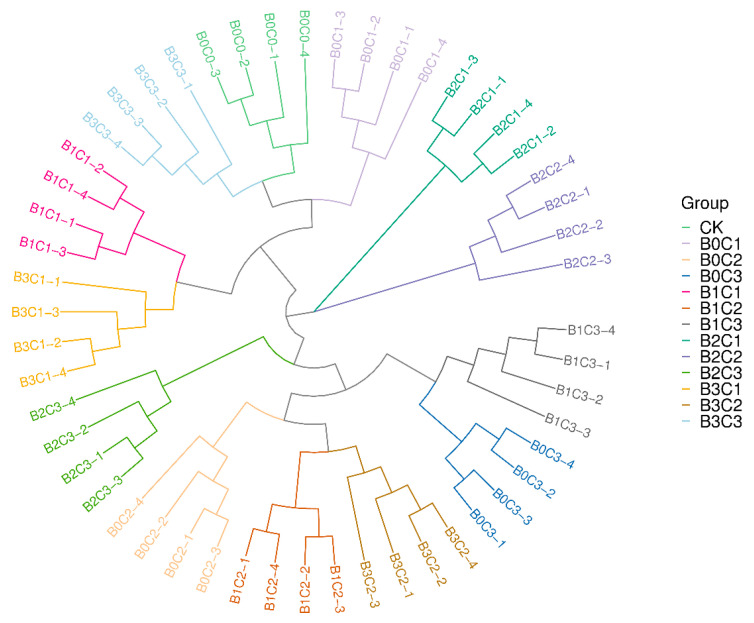
Clustering analysis based on Bray–Curtis. The closer the branch distance is, the more similar the samples are.

Sample hierarchical cluster analysis was used to evaluate the degree of similarity between samples. The cluster analysis results based on Bray–Curtis (Figure 5) show that the intra-group repetition under different treatments can be well aggregated, and the stratification between different treatments was apparent. This shows that the repeated samples within the group are highly similar, and the differences between groups are significant. According to the branch distance, the most similar treatments to CK were B0C1 and B0C2, and the sample with the greatest difference from CK was B3C1.

### 3.3. ANOVA Difference Statistics

**Figure 6:**
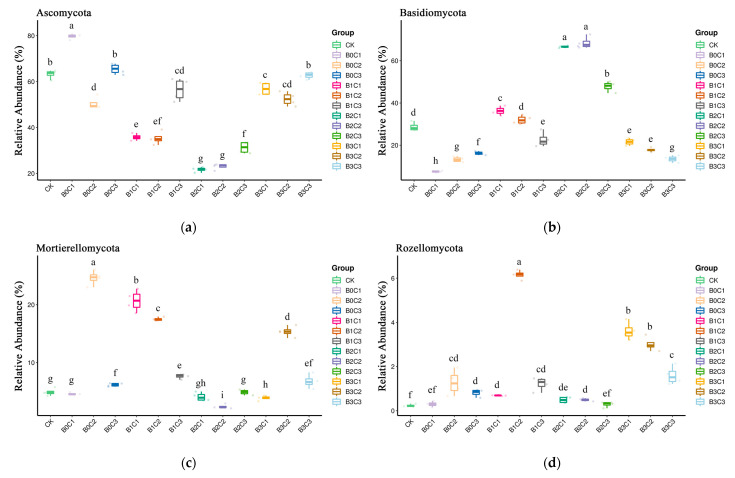

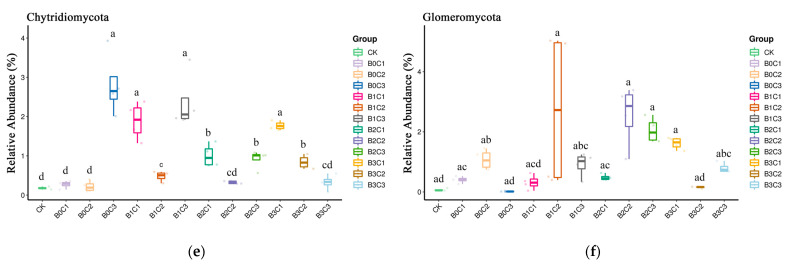
Boxplot diagram of different species at phylum level. (**a**): The species ranked first at the phylum level. (**b**): The species ranked second at the phylum level. (**c**): The species ranked third at the phylum level. (**d**): The species ranked fourth at the phylum level. (**e**): The species ranked fifth at the phylum level. (**f**): The species ranked sixth at the phylum level. (In the picture, a, b, c, d… indicate significant differences (*p* < 0.05) among treatments).

Statistical analysis was carried out at the phylum level. Different species were selected. The species whose relative abundance was more than 1% were analyzed by relative abundance boxplot analysis, and the abundance of dominant species within groups and between groups was obtained. Overall, the phase abundance of *Ascomycota* (Figure 6a) was between 21.70% and 79.76%, of which the relative abundance under the B0C1 treatment was the highest, at 79.67%, while that under the B2C1 treatment was the lowest, at 21.70%. *Ascomycota* decreased in all treatments with biochar addition and decreased significantly in all treatments except the B3C3 treatment. In the treatments without biochar, only the B0C2 treatment decreased *Ascomycota* significantly. The relative abundance of *Basidiomycota* (Figure 6b) was generally between 7.52% and 68.45%, with the highest relative abundance of 68.45% in the B2C2 treatment and the lowest relative abundance of 7.52% in the B0C1 treatment. In the treatments without biochar addition (B0) and the treatments with medium and high biochar addition level (B3), the relative abundance of *Basidiomycota* was significantly lower than that of the CK treatment. In the treatments with a medium biochar addition level (B2), the relative abundance of *Basidiomycota* was significantly higher than that of the CK treatment. The relative abundance of *Mortierellomycota* (Figure 6c) was generally between 2.39% and 24.65%, with the highest, of 24.65%, under the B0C2 treatment and the lowest, of 2.39%, under the B2C2 treatment. Among them, the relative abundance of *Mortierellomycota* increased significantly under the low biochar addition level (B1). The relative abundance of *Rozellomycota* (Figure 6d) and that of *Chytridiomycota* (Figure 6e) were the lowest in the CK treatment, while *Rozellomycota* relative abundance was the highest, at 6.15%, under the B1C2 treatment. The relative abundance of *Rozellomycota* was its highest, at 2.81%, under the B0C3 treatment. The relative abundance of *Glomeromycota* (Figure 6f) ranged between 0.02% and 2.55%, and the overall change was not significant with the addition of biochar and biological agents; the average relative abundance of *Glomeromycota* reached its maximum under the B2C2 treatment.

**Figure 7:**
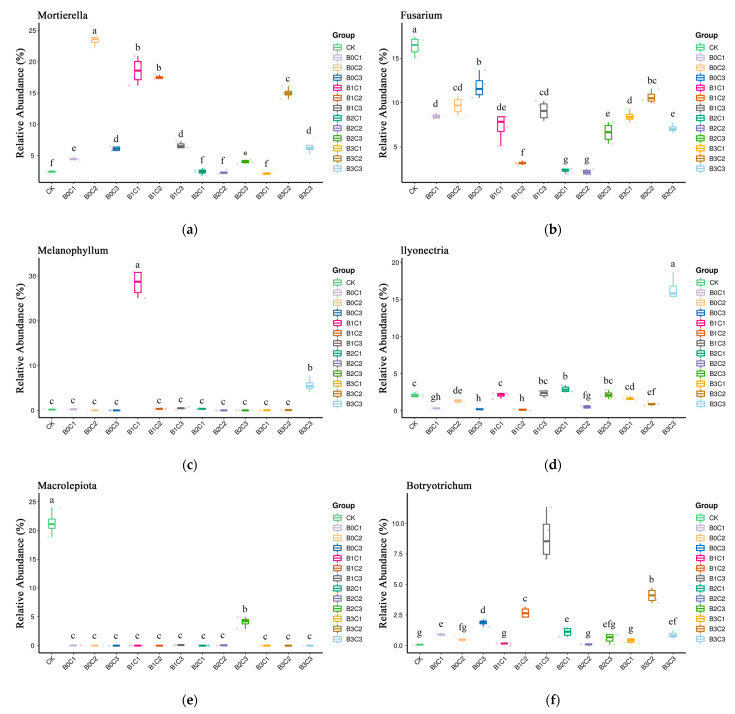

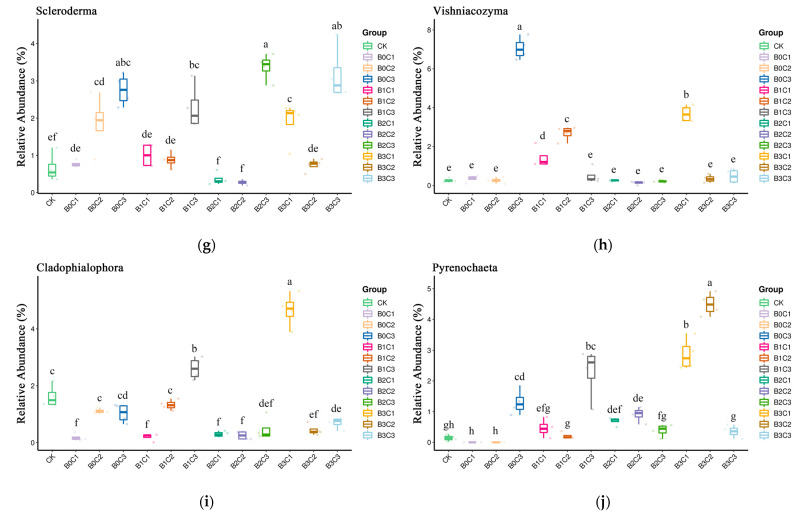
Boxplot diagram of different species at genus level. (**a**): The species ranked first at the genus level. (**b**): The species ranked second at the genus level. (**c**): The species ranked third at the genus level. (**d**): The species ranked fourth at the genus level. (**e**): The species ranked fifth at the genus level. (**f**): The species ranked sixth at the genus level. (**g**): The species ranked seventh at the genus level. (**h**): The species ranked eighth at the genus level. (**i**): The species ranked ninth at the genus level. (**j**): The species ranked tenth at the genus level. (In the picture, a, b, c, d… indicate significant differences (*p* < 0.05) among treatments).

Statistical analysis was carried out at the genus level, and the top 10 different species with relative abundance of more than 1% were selected for relative abundance boxplot analysis. For *Mortierella* (Figure 7a) the overall relative abundance was between 2.19% and 23.36%. It increased significantly in the treatments without biochar (B0) and low biochar (B1), and reached its maximum under the B0C2 treatment, of 23.36%. The relative abundance of *Fusarium* (Figure 7b) was generally between 2.16% and 16.36%, with the highest level, of 16.36%, in the CK treatment. With the addition of biochar and biological agents, the relative abundance of *Fusarium* decreased significantly and was the lowest under B2C2 treatment, at 2.16%. *Melanophyllum* was generally relatively abundant (Figure 7c) and was not even detected in some processes, such as in B2C2 and B2C3 processing. Of all of the treatments, only more than 1% were processed by B1C1 and B3B3, which were 28.35% and 5.68%, respectively. The relative abundance of *Ilyonectria* (Figure 7d) generally ranged between 0.13% and 16.45% under B1C2 and B3C3 treatments, respectively. In the treatments without biochar addition (B0), the relative abundance of *Ilyonectria* decreased significantly. In the treatments with biochar addition, the relative abundance of *Ilyonectria* increased significantly under the B2C1 and B3C3 treatments and decreased significantly under the B1C2, B2C2 and B3C2 treatments. The relative abundance of *Macrolepiota* (Figure 7e) was only more than 1% in the CK treatment and B2C3 treatment, at 21.28% and 4.05%, respectively. It was not more than 1% under the rest of the treatments, and even no *Macrolepiota* was detected under individual treatments such as B0C3, B1C1, B1C2 and B3C2. The relative abundance of *Botryotrichum* (Figure 7f) generally ranged from a low of 0.07% under the CK treatment to a high of 8.86% under the B1C3 treatment. The relative abundance of *Scleroderma* (Figure 7g) was between 0.28% and 3.38%. With the different addition levels of biochar and biological agents, the relative abundance of *Scleroderma* was also different. Under the B2C1 and B2C2 treatments, its relative abundance decreased, but the decrease was not significant. In other treatments, the relative abundance of *Scleroderma* increased and reached its maximum under the B2C3 treatment. The relative abundance of *Vishniacozyma* (Figure 7h) was generally between 0.13% and 7.05%. Under the B0C3, B1C1, B1C2 and B3C1 treatments, it was significantly higher than that under the CK treatment, and was highest under the B0C3 treatment. The relative abundance of *Vishniacozyma* did not reach a significant level under other treatments. The relative abundance of *Cladophialophora* (Figure 7i) was generally between 0.18% and 4.66%. Compared with the CK treatment, it increased significantly under the B1C3 and B3C1 treatments, reaching 4.66% under the B3C1 treatment. The relative abundance of *Cladophialophora* decreased significantly under the B0C1, B1C1, B2C1, B2C2, B2C3, B3C2 and B3C3 treatments, measuring 0.18% under the B0C1 treatment. The relative abundance of *Pyrenochaeta* (Figure 7j) was 0.14% in the CK treatment. *Pyrenochaet* was not detected in the B0C1 and B0C2 treatments. The relative abundance of *Pyrenochaeta* increased significantly under the B0C3, B1C3, B2C1, B2C2, B3C1 and B3C2 treatments, reaching 4.49% under the B3C2 treatment, the highest among all treatments.

### 3.4. Random Forest Analysis

**Figure 8:**
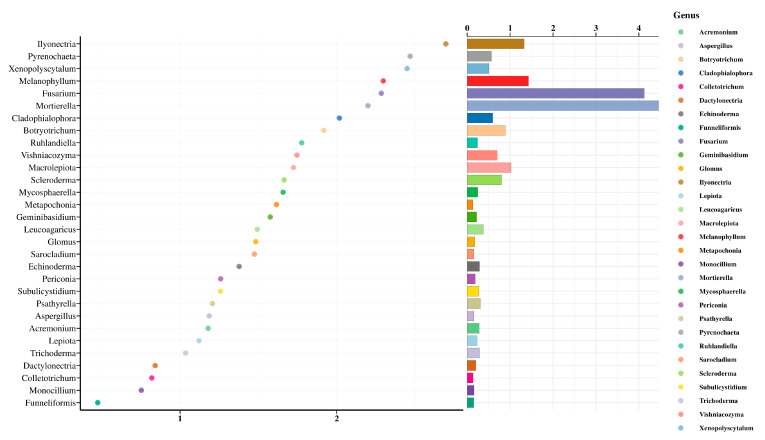
Results of random forest analysis. On the left is the point map of species importance. The abscissa is the measure of importance, and the ordinate is the species name sorted by importance. On the right is the relative abundance bar chart of the species.

In order to effectively and accurately classify fungal communities in soil, the key species with differences between groups must be found. We performed random forest analysis, taking the top 30 species by relative abundance at the genus level, and used R-packet randomForest to draw the species importance point map. On the surface of the analysis results (Figure 8), *Ilyonectria*, *Pyrenochaeta* and *Xenopolyscytalum* were the top three critical species in the soil, and their MeanDecreaseGini values were 2.70, 2.50 and 2.45, respectively. The results of the relative abundance histogram on the right showed that the top three species by relative abundance were *Mortierella*, *Fusarium* and *Melanophyllum*, respectively.

## 4. Discussion

Sequence analysis of the fungal ITS1 region provided detailed information on fungal community structure in the rhizosphere soil of *Panax notoginseng* [28]. Understanding the fungal structure in the rhizosphere soil of continuous cropping *Panax notoginseng* is very important for the field management of *Panax notoginseng*. At the gate level, different treatments did not change the dominant gate in the rhizosphere soil of *Panax notoginseng*. *Ascomycota*, *Basidiomycota* and *Mortierellomycota* were still the dominant gates in the soil, a finding similar to the results of previous studies [29,30]. In previous reports, *Fusarium*, *Cylindrocarpon*, *Ilyonectria* and other pathogenic bacteria were the dominant bacteria in the rhizosphere soil of perennial *Panax notoginseng* [31,32]. In our experimental results, the top five dominant fungi in the rhizosphere soil of *Panax notoginseng* were *Macrolepiota*, *Fusarium*, *Mortierella*, *Ilyonectria* and *Scleroderma*. Except for *Cylindrocarpon*, the experimental results were similar to those of previous studies. The rotation cycle of *Panax notoginseng* is generally longer than six years, because with the increase in planting years, the diversity of fungi in the soil increases, so that the soil environment is no longer suitable for the growth of *Panax notoginseng*. The results of Tan et al. also showed that with the increase in planting time, the ASVs and the Chao1 and Shannon indices in the rhizosphere soil of *Panax notoginseng* increased significantly [14]. Our experimental results showed that the number of ASVs and the Chao1 and Shannon indices increased significantly with the addition of biochar and *Bacillus subtilis*, which indicated that the fungal community in the rhizosphere soil of *Panax notoginseng* became rich after adding biochar and *Bacillus subtilis*. This may be because adding biochar improves the soil environment and provides a suitable environment for the survival and reproduction of soil proto fungi. Therefore, the effect of biochar addition on the soil health of *Panax notoginseng* needs to be further verified.

It has been confirmed in previous studies that biochar has strong stability [33], and after application to soil, it can have an effect on soil bulk density, water content and cation exchange capacity [34]. At the same time, base ions such as Ca^2+^, K^+^ and Mg^2+^ in the biochar will be released to a certain extent after entering the soil, which can increase the base saturation and adjust the pH value of the soil [35,36]. Biochar itself contains a wealth of functional groups that increase the total soil charge and soil cation exchange capacity after entering the soil [37]. Some scholars believe that the surface oxidation capacity and surface cation adsorption capacity of biochar may be the main reasons for the increase in soil cation exchange capacity [38]. The effect of biochar on the soil’s physical and chemical properties will directly or indirectly affect the microorganisms in the soil. Previous studies have shown that biochar can improve soil biological properties by enhancing the function of soil microorganisms and changing community structure [39,40]. Our experimental results showed that the Chao1 index and Shannon index in the rhizosphere soil of *Panax notoginseng* increased significantly with the increase in biochar application at the same level of biological agent addition. This showed that with the increase in the amount of biochar, the richness and evenness of the fungal community structure in the rhizosphere soil of *Panax notoginseng* were improved. This result may be caused by a variety of factors; first of all, the highly porous structure of biochar can provide a habitat for microorganisms, which protects the non-dominant colonies in the soil to a certain extent. Secondly, the different materials for preparing biochar and the different soils tested finally led to the change in fungal community diversity in the rhizosphere soil of *Panax notoginseng*. More importantly, the amount of biochar application is an essential reason for the difference in fungal diversity in the rhizosphere soil of *Panax notoginseng* [41]. Only when the amount of biochar application is high enough to significantly change the soil moisture content, pH conditions and nutrient concentration can lead to significant changes in soil microbial diversity and composition [42]. The experimental results also showed that under low-level biological agent addition (C1), the Chao1 index and Shannon index did not increase significantly at low (B1) and medium (B2) biochar addition levels, but only at the high biochar addition level (B3).

Many microorganisms can promote plant growth and have beneficial effects on the root system and the growth of the whole plant growth as part of the plant rhizosphere. These bacteria were designated as plant growth-promoting rhizobacteria (PGPRs). PGPRs promote plant growth mainly by improving plant tolerance to abiotic stress, immobilizing nutrients absorbed by plants, and producing plant growth regulators, iron carriers and volatile organic compounds. In recent years, using microbial agents as biocontrol agents has effectively inhibited plant pathogens. *Bacillus subtilis* has been proven to be an excellent biocontrol bacterium. It has been confirmed in previous experiments that inoculating *Bacillus subtilis* into the rhizosphere of diseased Chinese cabbage can effectively inhibit the abundance of pathogens [43]. Previous studies have shown that after *Bacillus subtilis* inoculation, the abundance of *Fusarium* in soil decreased significantly, while the mortality and prevalence rate of *Panax notoginseng* were significantly improved [44], indicating that *Bacillus subtilis* can increase production and improve the quality of *Panax notoginseng*. We also reached the same conclusion in our experiment: With the addition of *Bacillus subtilis* biological agents, the abundance of *Fusarium* in the rhizosphere soil of *Panax notoginseng* decreased significantly. Under the CK treatment, the relative abundance of *Fusarium* in the rhizosphere soil of *Panax notoginseng* was the highest, which was consistent with the distribution characteristics of pathogenic bacteria in the rhizosphere soil of perennial *Panax notoginseng*. The overall change in the relative abundance of *Ilyonectria* was similar to that of *Fusarium*, However, it increased slightly in individual treatments but not significantly, possibly due to the uneven distribution of colonies in the soil. *Cylindrocarpon* is the main pathogen causing *Panax notoginseng* rust spots [45]. In the course of our experiment, there were no large-scale rust spots. Therefore, although *Cylindrocarpon* was also one of the pathogenic bacteria in the rhizosphere soil of *Panax notoginseng*, it was not detected in our experiment.

Biofilm is an aggregate of organized growth of microorganisms [46]. Bacteria attach to the surface of inert or active bodies, propagate, differentiate, and secrete some polysaccharide matrix, which wraps the bacterial community to form a bacterial aggregate membrane [47]. Biofilm can resist adverse environmental factors and enhance and stabilize the metabolic activity of microorganisms to ensure growth and reproduction in an unstable environment [48]. In previous studies, biochar could capture a large number of *Bacillus subtilis* bacteria to form biofilm, which increased the survival of beneficial microorganisms [49]. In the cultivation of hot pepper, the addition of biochar can not only retain its beneficial effect on soil physical and chemical properties, but also improve the colonization effect of *Bacillus subtilis* [50]. Our experimental results showed that the relative abundance of *Fusarium* in the treatments without biochar was higher than that in the treatments with biochar but lower than that in the CK treatment. This shows that biochar provides a suitable environment for colonization and can enhance the inhibitory effect of *Bacillus subtilis* on *Fusarium* in the rhizosphere soil of *Panax notoginseng*. We can reasonably speculate that this was due to the fact that *Bacillus subtilis* can quickly form a stable biofilm on biochar after entering the soil.

## 5. Conclusions

Our experiments confirmed that the combined application of biochar and *Bacillus subtilis* increased the diversity of fungi in the rhizosphere of *Panax notoginseng*. This is because biochar provides a suitable growth environment for soil proto fungi after being applied to the soil. The addition of biochar and the *Bacillus subtilis* preparation had a significant reducing effect on pathogenic fungi in the rhizosphere soil of *Panax notoginseng*, among which *Fusarium* decreased by 35.02% and 86.79%. The B2C2 treatment had the best inhibitory effect on *Fusarium*, indicating that the dose of biochar and *Bacillus* subtilis preparation should be 110 kg·hm^−2^ and 15 kg·hm^−2^, respectively, in the areas with frequent occurrence of acute bacterial wilt and root rot. The combined application of biochar and *Bacillus* subtilis showed an inhibitory effect on *Ilyonectria* on the whole. For the areas with frequent occurrence of yellow rot root rot, the application amount of biochar should be 80 kg·hm^−2^, and that of *Bacillus subtilis* should be 15 kg·hm^−2^.

With the application of biochar and *Bacillus subtilis* preparation, the pathogenic fungi in the rhizosphere soil of *Panax notoginseng* were effectively reduced, and the fungal structure was also stable. Therefore, in the long-term planting process, the long-term effects of biochar and *Bacillus subtilis* on the rhizosphere fungal community of *Panax notoginseng* need to be further studied.

## Figures and Tables

**Table 1:** Applied amounts of biochar and biological agents.

Treatment	Applied Amount of Biochar (kg·hm^−2^)	Applied Amount of Biological Agents (kg·hm^−2^)
CK	0	0
B0C1	0	10
B0C2	0	15
B0C3	0	25
B1C1	80	10
B1C2	80	15
B1C3	80	25
B2C1	110	10
B2C2	110	15
B2C3	110	25
B3C1	140	10
B3C2	140	15
B3C3	140	25

**Table 2:** Alpha diversity index of fungi in rhizosphere soil of *Panax notoginseng* (a, b, c, d… indicate significant differences (*p* < 0.05) among treatments based on Duncan’s mean test).

Treatment	ASVs	Chao1	Shannon	Simpson	ACE	Good_Coverage
CK	189.00 ± 9.87c	189.06 ± 9.85c	4.08 ± 0.06f	0.85 ± 0.01h	188.73 ± 10.06c	0.99
B0C1	199.73 ± 7.01c	199.95 ± 7.13c	3.81 ± 0.08g	0.79 ± 0.01i	199.79 ± 6.69c	0.99
B0C2	180.00 ± 17.07c	180.03 ± 17.10c	5.58 ± 0.12c	0.94 ± 0.01e	179.82 ± 16.88c	0.99
B0C3	316.98 ± 10.64b	316.98 ± 10.64b	6.16 ± 0.10b	0.96 ± 0.00b	316.60 ± 10.04c	0.99
B1C1	166.18 ± 26.29c	166.18 ± 26.29c	4.68 ± 0.11d	0.92 ± 0.01f	166.55 ± 26.53c	0.99
B1C2	370.00 ± 4.06a	370.19 ± 4.02a	6.00 ± 0.02b	0.95 ± 0.00d	370.14 ± 3.71a	0.99
B1C3	259.48 ± 54.81bc	259.50 ± 54.80bc	6.53 ± 0.41ab	0.98 ± 0.00a	259.42 ± 54.78bc	0.99
B2C1	242.75 ± 3.90c	242.79 ± 3.92c	4.37 ± 0.08e	0.86 ± 0.01h	242.33 ± 4.71c	0.99
B2C2	215.00 ± 29.66c	215.01 ± 29.66c	3.96 ± 0.19fg	0.77 ± 0.02i	214.95 ± 29.56c	0.99
B2C3	229.50 ± 6.02c	229.51 ± 6.00c	5.93 ± 0.04b	0.96 ± 0.00b	229.31 ± 6.31c	0.99
B3C1	324.00 ± 27.24b	324.00 ± 27.24b	6.62 ± 0.09a	0.97 ± 0.00a	323.91 ± 27.39b	0.99
B3C2	330.25 ± 19.90b	330.27 ± 19.91b	6.08 ± 0.07b	0.96 ± 0.00c	330.69 ± 20.17b	0.99
B3C3	170.50 ± 22.60c	170.50 ± 22.60c	4.67 ± 0.13a	0.90 ± 0.01g	170.91 ± 22.88c	0.99

ASV is Amplicon sequence variant (amplified subsequence variation). Each de-duplicated feature sequence produced by quality control operations such as denoising and splicing by DADA2 method. The abundance table of these sequences in the sample is called feature table (feature-table); Chao index: The actual number of species in the community was estimated by calculating the ASV of only one or two reads in the community; Shannon index: the higher the species diversity is, the more uniform the species distribution is, and the larger the Shannon index value is (which can reflect both the species richness and the evenness of distribution); Simpson index: the probability that two randomly selected individuals belong to different species. The higher the Simpson index, the higher the community diversity; Ace index: an index used to estimate the number of ASVs in a community; good_coverage index: this index reflects the sequencing depth, and the closer the index is to 1, the stronger the indication that the sequencing depth has basically covered all the species in the sample.

## Data Availability

The data are contained within the article.
